# Lack of association among *TNF*-α gene expression, -308 polymorphism (G > A) and virulence markers of *Helicobacter pylori*

**DOI:** 10.1186/s40409-015-0054-3

**Published:** 2015-12-30

**Authors:** Luanna Munhoz Zabaglia, Mariane Avante Ferraz, Weendelly Nayara Pereira, Wilson Aparecido Orcini, Roger Willian de Labio, Agostinho Caleman Neto, Fernanda Wisnieski, Juliana Garcia de Oliveira, Marilia de Arruda Cardoso Smith, Spencer Luiz Marques Payão, Lucas Trevizani Rasmussen

**Affiliations:** Universidade Sagrado Coração, Rua Irmã Arminda 10-50, Jardim Brasil, CEP 17011-160 Bauru, SP Brazil; Faculdade de Medicina de Marília, Rua Lourival Freire 240, 17519-050 Marília, SP Brazil; Universidade Federal de São Paulo, Rua Sena Madureira, 1500, 04021-001 São Paulo, SP Brazil

**Keywords:** *H. pylori*, *TNF*-α, Gene expression, Gastric cancer, *cag*A

## Abstract

**Background:**

Tumor necrosis factor plays a critical role in the pathogenesis of gastric diseases such as gastric cancer, and an abnormal inflammatory response has frequently been observed in dyspeptic patients. *Helicobacter pylori* infection can induce a gastric mucosal inflammatory response that may be influenced by -308 (G > A) polymorphisms and gene expression of the *TNF*-α gene.

**Methods:**

One hundred and thirty-four gastric biopsy samples were collected from patients of both genders (61♂ and 73♀, mean age 40.3 ± 24.2 years) with gastric symptoms. The -308 (G > A) polymorphism of *TNF*-α was characterized using polymerase chain reaction (PCR) and restriction fragment length polymorphism (RFLP). The expression level was measured using real-time PCR, and relative quantification (RQ) was calculated using the comparative CT method (2^-ΔΔCT^).

**Results:**

The analysis revealed an increase in *TNF*-α gene expression in patients with gastritis; on the other hand, no statistical differences were observed in patients with gastric cancer. In addition, no association was found among -308 polymorphism genotypes, virulence markers, or *TNF*-α gene expression.

**Conclusions:**

*Helicobacter pylori* induces a large increase in *TNF*-*α* expression in patients with gastritis, regardless of tissue inflammation, but after the tissue becomes neoplastic, the presence of bacteria did not influence expression. These results suggest that the TNF-*α* pathway may play an important role in the progression from gastritis to gastric cancer

## Background

*Helicobacter pylori* (*H. pylori*) is a gram-negative bacterium that colonizes more than half of the world population, causing chronic infection. It comprises a major risk factor for gastric cancer (GC), which is the fourth most commonly diagnosed cancer and the second most deadly one, with over 750,000 new cases annually worldwide [1, 2].

Several factors are correlated with the severity of clinical outcomes. These factors include the virulence markers of *H. pylori,* such as the genes *cag*A, *vac*A and *dup*A, and host genetic factors. However, bacterial factors or host genetic factors alone are not sufficient to explain why so few individuals harboring *H. pylori* develop gastric disease [3, 4].

Evidence suggests that the main pathophysiological event in the *H. pylori* infection of gastric mucosa is the induction of a gastric mucosal inflammatory response. This reaction is induced by the contact of *H. pylori* with gastric cells, and is followed by neutrophil and mononuclear cell activation and the transcription and synthesis of pro-inflammatory and anti-inflammatory cytokines [4, 5].

Tumor necrosis factor alpha (TNF-α) is a very important multifunctional pro-inflammatory cytokine. It provides a rapid form of host defense against infection by gram-negative bacteria. It is produced by activated monocytes and macrophages, which play a key role in inflammatory response [6]. However, this cytokine is also associated with gastric acid inhibition and paracrine effects, both of which may increase the risk of developing gastric cancers [7, 8].

Several polymorphisms in the promoter region of the *TNF*-α gene have been associated with a variety of inflammatory diseases and cancer [9, 10]. *TNF*-α-308 G > A (rs1800620), one of the most frequently studied polymorphisms on this gene, seems to have an influence on transcriptional activities and may contribute to the development of gastric disease [11]. Furthermore, previous studies showed that *H. pylori* virulence markers, such as *cag*A, *vac*A, and *dup*A genes, play important roles in gastric mucosal inflammation and injury through activated inflammatory cell infiltration [12–14].

CagA-cytotoxin is a virulence factor that has been well established as being associated with the development of peptic ulceration or gastric adenocarcinoma. This protein is translocated into the epithelial cytosol, thus causing changes that favor the persistence of the bacterium, but which also lead to changes in gastric cells without the direct adherence of bacteria in the stomach [15].

Another important virulence marker is vacuolating cytotoxin A (*vac*A), which encodes the secreted toxin VacA. The VacA toxin exhibits multiple cellular activities, and all *H. pylori* strains possess a *vacA* gene. This gene displays allelic diversity in two main regions: the signal (s) region (s1 and s2) and the middle (m) region (m1 and m2). Different combinations are associated with vacuolating capabilities. In addition, *vac*A s1/m1 strains are correlated with greater vacuolating activity and gastric disease, while *vac*A s2/m2 strains appear to be nonvacuolating [16–18].

Thus, the absence of an association with *H. pylori* positivity is understandable, as is the strong association between *cagA* and s1/m1 of *vac*A factors, and as well as their association with gastritis and gastric cancer. The secretion of the vacuolating toxin vacA has been found to induce vacuole formation in the gastric epithelial cellular membrane, as well as the modulation of apoptosis rather than the secretion of inflammatory cytokines. These mechanisms, in turn, influence gastric carcinogenesis [19].

The strong association between the inflammatory process and gastric carcinogenesis is very important in the evaluation of genetic polymorphisms in cytokine genes. The polymorphism in the *TNF* gene promoter region may influence not only cytokine gene expression, but also susceptibility to disease and pathogenesis [20]. Furthermore, the relationship among genes, host cytokine polymorphisms, their expression, and the severity of clinical outcomes in the ethnically diverse Brazilian population needs to be further characterized.

We believe that the relationship between the host genetic factors and the genetic factors of *H. pylori* can determine the severity of gastric disease in infected individuals. Thus, the aims of the current study were to determine the expression levels of the *TNF*-α cytokine gene in tissues samples from gastric cancer patients, chronic gastritis patients and uninfected controls, to evaluate the *TNF*-α -308 G > A polymorphism, and to determine the influence of *H. pylori*, *cag*A, *vac*A and *dup*A virulence markers on gastric carcinogenesis.

## Methods

This study was approved by the Research Ethics Committee of Sacred Heart University (Universidade do Sagrado Coração) in Bauru, São Paulo, Brazil (under registration n. 068/12). Written informed consent was obtained from every patient involved in the study. All tissue samples were obtained by means of endoscopy or gastric surgery, procedures which were performed in the Department of Gastroenterology at Marília Medical School, the São Paulo Hospital, or the João de Barros Barreto University Hospital.

### Study population

One hundred and thirty-four gastric biopsy samples were collected from patients of both genders (61♂/73♀, mean age 40.3 ± 24.2 years) with gastric symptoms. Histological parameters were analyzed and revealed 70 patients with chronic gastritis (G), 40 with normal gastric tissue (C), and 24 with gastric cancer (GC) tissue. The histological parameters were classified according to the Sydney system [21] and Lauren’s classification [22]. Patients exposed to chemotherapy or radiotherapy before surgery or those who had received antimicrobial therapy, anti-cholinergic and anti-inflammatory agents, or proton pump inhibitors up to 30 days before endoscopy were excluded.

### DNA extraction and *H. Pylori* status

DNA from gastric biopsies was extracted using the QIAamp® tissue kit (Qiagen, Germany) according to the manufacturer’s recommendations. The presence of *H. pylori* and virulence markers from *cagA*, *vacA* and *dupA* genes were all detected using polymerase chain reaction, as in previous studies [23–27].

### Genotyping of *TNF*-α

The -308 (G > A) polymorphism of *TNF*-α was characterized using both polymerase chain reaction (PCR) and the restriction fragment length polymorphism (RFLP) technique according to the Wilson’s protocol [28].The 107 base pair fragment was amplified using the primers ^5′^AGGCAATAGGTTTTGAGGGCCAT^3^′ and ^5′^TCCTCCCTGCTCCGATTCCG^3′^, and the amplification condition that was utilized included 35 cycles: 45 s at 94 °C, 45 s at 61 °C, and 45 s at 72 °C. The amplicons were digested with *Nco*I (Fermentas, USA) overnight at 37 °C, visualized in 3 % agarose gel, stained with ethidium bromide, and analyzed using an Alpha Imager 2200 (Alpha Innotech Corporation™). The fragments visualized were 107 bp in length in the case of allele A and 87 and 20 bp in length in the case of allele G.

### RNA Extraction and cDNA Synthesis

After collection, the biopsy fragments were immediately placed into an RNAlater solution (Applied Biosystems™, USA) and stored at -20 °C until use. Total RNA was isolated using an RNeasy Mini Kit (Qiagen, Germany), and RNA concentration and quality were measured using the NanoDrop 2000 Spectrophotometer (NanoDrop, USA). The concentration was adjusted to 500 ng, and cDNA synthesis was conducted using High-Capacity cDNA Reverse Transcription Kits (Applied Biosystems™, USA) following the manufacturer’s instructions.

### Quantitative analysis of *TNF*-α gene expression

In order to determine *TNF*-α gene expression (Hs01113624_g1), the normal gastric tissue sample, which was negative for *H. pylori* infection, was designated as a calibrator. All reactions were repeated, and *GUSB* (Hs00187320_m1), *UBC* (Hs00221499_m1), and *TBP* (Hs00187332_m1) were used as reference genes.

The expression level was measured using the TaqMan® System (Life Technologies, USA) within the Applied Biosystems 7500 Fast Real-Time PCR system (Applied Biosystems™, USA). Relative quantification (RQ) was calculated using the comparative CT method (2^-ΔΔ^ct).

### Statistical analysis

The *χ*^2^ test was used to compare the *TNF*-α allele and genotype distributions. Odds rates (ORs) were calculated using a dominant model (i.e., combining heterozygotes and homozygotes for the minor allele). To analyze gene expression, the two-tailed Student’s *t*-test was used. Statistical analyses were performed using the GraphPad Prism 5 statistical package. Statistical significance was accepted at *p* < 0.05.

## Results

### *H. Pylori* infection and virulence markers

Table 1 summarizes the characteristics of the 134 study subjects, 71 (53 %) of whom tested positive for *H. pylori* infection and 63 (47 %) of whom tested negative for *H. pylori* infection. *H. pylori* presence was associated with the development of G and GC (*p* < 0.001), and an association between *cagA* and s1/m1 alleles of the *vacA* gene in samples from G and GC was observed (*p* < 0.001).

**Table 1:** Presence and characteristics of *Helicobacter pylori* in gastric biopsies

Histolohy (*N*/%)	*Positive samples (n%)*	*cagA+ (%)*	*dupA+ (%)*	*vacA (%)*		
				*s1/m1*	*s2/m2*	*s1/m2*
Control (40/100)	3 (7.5)	1 (2.5)	1 (2.5)	1 (2.5)	2 (5)	–
Chronic gastritis (70/100)	47 (67.1)	19* (27.1)	25 (35.7)	19* (27.1)	21 (30)	7 (10)
Gastric cancer (24/100)	21 (87.5)	20* (83.3)	15 (62.5)	21* (87.5)	–	–
Total (134)	71	40	41	41	23	7

*N*: number of the total of samples; *n*: number of positive samples; *: statistical difference when *p* < 0.05

For the other analyses, the group with gastric cancer tissue that also tested negative for *H. pylori* was excluded due to the low sample number.

### *TNF*-α-308 (G > a) polymorphism

Genotype and allele frequencies are presented in Table 2. With the exception of the gastritis group (*p* = 0.076), genotype distributions were not within the Hardy-Weinberg equilibrium in the gastric cancer group (*p* = 0.012) or in the control group (*p* = 0.0065). Statistical analysis did not detect an association between the polymorphisms and gastric disease or between the polymorphisms and the presence of *H. pylori.*

**Table 2:** Genotype and allele frequencies of −308 G *>* A polymorphism of the *TNF-α* gene, adjusted for the presence of. *H pylori* in patients with normal gastric mucosa, gastritis and gastric cancer

	Normal (*n* = 40)		Chronic gastritis (*n* = 70)			Gastric cancer (*n* = 24)		
	*Hp + (n: 03)*	*Hp - (n: 37)*	*Hp + (n: 47)*	*Hp - (n: 23)*	*OR (95 % CI), p*	*Hp + (n: 21)*	*Hp - (n: 03)*	*OR (95 % CI, p*
Genotypes								
* G/G*	03 (100 %)	30 (81 %)	34 (72.3 %)	18 (78.3 %)	1.55 (0.48-4.98), 0.46	15 (71.4 %)	02 (66.6 %)	3.66 (0.48-27.85), 0.2
* G > A*	–	04 (10.9 %)	09 (19.1 %)	05 (21.7 %)		03 (14.3 %)	01 (33.4 %)	
* A/A*	–	03 (8.1 %)	04 (8.6 %)	–		03 (14.3 %)	–	
Alleles								
* G*	6 (100 %)	64 (86.5 %)	77 (82 %)	41 (89 %)	1.36 (0.61- 3.03), 0.55	33 (78.5 %)	05 (83.3 %)	1.84 (0.70- 4.81),021
* A*	–	10 (13.5 %)	17 (18 %)	05 (11 %)		09 (21.5 %)	01 (16.7 %)	

Hp: *Helicobacter pylori*; n: number of samples

### *TNF*-α expression in patients with gastritis and gastric cancer

It is important to note that, in the expression analysis, samples from all of the test groups were compared to the normal gastric tissue samples, which were negative for *H. pylori* infection (control group RQ = 1.072)*.*

The expression analysis revealed increased expression in patients with gastritis (RQ: 2.912) in relation to the control group (*p* = 0.0001). In the second analysis, the gastritis group was divided according to the presence of *H. pylori*: *TNF*-α expression was significantly higher in patients with gastritis who had tested positive for *H. pylori* (*p* = 0.0001; RQ = 3.774) (Table 3). However, no statistical difference was observed in patients with gastritis and without *H. pylori* (*p* = 0.3697; RQ: 1.297) (Table 3).

**Table 3:** Comparing the mRNA expression between control and case groups

Gene	Histology analyses		*n*	RQ Mean ± SD	*p*
	Control group (*n* = 37)	Case groups			
TNF-α	Normal gastric tissue *Hp-* *(RQ: 1.072* ± 0.45*)*	Gastritis	70	2.912 ± 2.03	0.0001*
		Gastritis *Hp+*	47	3.774 ± 1.98	0.0001*
		Gastritis *Hp-*	23	1.297 ± 0.89	0.3697
		Neoplasic *Hp+*	21	1.163 ± 1.09	0.6332

Hp: *Helicobacter pylori*; n: number of samples; RQ: relative quantification; SD: standard deviation; *: statistical difference when *p* < 0.05

Conversely, there was no statistical difference observed when the control group (RQ = 1.072) was compared to patients with gastric cancer. Expression in neoplastic tissue (RQ = 1.163; *p* = 0.6332) did not present any alterations (Table 3).

### Correlation among *TNF*-α gene expression, -308 (G > a) polymorphism, and *H. Pylori* virulence markers

No association was observed between the genotypes or alleles of the -*308 (G > A)* polymorphism and *TNF*-α gene expression in any of thesamples. First, gene expression of the samples withG/G genotypes was compared to that ofG > A + A/A genotypes in patients with gastritis (*p* = 0.8269) and with gastric cancer (*p* = ns).The small number of A/A genotypes,particularly in the gastric cancer samples, may have influenced theanalysis. In subsequent analyses, gene expression was compared tothe presence of *cag*A gene (*p* = 0.4677), to the presence of the*dup*A gene (*p* = 0.0518), and to genotypes s1/m1 and s2/m2 of the *vac*A gene (*p* = 0.3578). The presence of more pathogenic of *H. pylori*strains did not induce changes in *TNF*-α expression. In addition, *cagA*, *vacA* and *dupA* genes were not found to be associated with differential gene expression of *TNF*-α cytokine.

## Discussion

*H. pylori* infection in gastric mucosa causes an inflammatory response in the host characterized by infiltration of immune cells such as macrophages, neutrophils, and lymphocytes. This reaction is most likely induced by the contact of the bacterium with the epithelium; however, it has been estimated that only 20 % is adhered to the gastric mucosa and therefore able to activate the inflammatory response [29].

Thus, it is evident that virulence factors play a more important role in the progression of gastric carcinogenesis than the mere presence of the bacteria does.

The *TNF*-α cytokine plays an essential role in host immunity against infectious disease and cancer progression [30]. However, studies on the association between *TNF*-α-308 G > A polymorphisms and gastric carcinogenesis are very controversial [31–35].

Li et al. [6] evaluated several studies on the *TNF*-α-308 G > A polymorphism in different ethnic populations. In Caucasian populations, they demonstrated a weak association between the *TNF-α*-308 A variant and gastric cancer risk. Meanwhile, no associations were found between this A variant and gastric carcinogenesis in East Asian samples or other ethnic populations, including the Brazilian population. Two of the most recent Brazilian articles cited in this meta-analysis evaluated patients with chronic gastritis and gastric cancer and did not find any association between the *TNF-α*-308 A polymorphism and gastric carcinogenesis [4, 36].

The association between inflammation and disease progression is well described in a gastric cancer model, which begins with chronic gastritis that persists and may induce changes such as metaplasia, dysplasia and, finally, cancer [37]. We also observed that, in patients with chronic gastritis, *TNF-α* mRNA was overexpressed in relation to the control patients, and the association was greater among patients who had gastritis and were also *H. pylori* positive. On the other hand, there was no increased expression in patients with gastric cancer. These results indicate the involvement of this cytokine in initial carcinogenesis. A recent study with a mouse model showed that *TNF*-α is also produced in excess in early colonic lesions in cases of colorectal cancer, and the data supported the closest association between *TNF*-α and early tumor progression [38].

Bartchewsky et al. [39] showed that patients with chronic gastritis infected by *cag*A-positive strains of *H. pylori* had the highest cytokine mRNA and protein levels. However, we did not find an association between higher cytokine mRNA level and patients infected by *cag*A-positive strains. Santos et al. [4] found similar results regarding the presence of the *TNF*-α-308 G > A polymorphism and infection by cagA-positive strains. This polymorphism apparently does not affect the presence of *H. pylori* or the progression of gastric disease.

More recently, Caliskan et al. [40] performed a study to determine the relationship between among *H. pylori* genotypes and their effects on cytokine release levels. These authors studied five genotypes: *cag*A and *vac*A s1m2 (genotype 1); *cag*A and *vac*A s1m1 (genotype 2); *cag*A, *vac*A s1m2, and *bab*A2 (genotype 3); *vac*A s2m2 (genotype 4); and *cag*A and *vac*A s2m2 (genotype 5). These researchers found that all of these genotypes increased IL-1β, IL-6, IL-8, IL 10, and *TNF*-α levels in THP-1 cells. However, genotype 5 was found to cause higher amounts of IL-1β, IL-6, *TNF*-α, and IL-10, whereas genotype 1 induced the highest levels of IL-8. Thus, they suggested that the host cytokine response varies depending on the individual specific immune response to different *H. pylori* strains.

In another recent study, Michalkiewicz et al. [41] analyzed gene expression of innate immunity and cytokines in the gastric mucosa from *H. pylori*-infected children and uninfected children and found high mRNA expression of IL-6, IL-10, IFN-γ, and *TNF*-α. However, *H. pylori cag*A-positive strains were associated only with higher IL-6 and IL-10 mRNA expression in relation to the *cag*A-negative strains. Meanwhile, *TNF*-α presented increased protein levels and was correlated with bacterial density.

Our results are consistent with those of several authors, and we offer two hypotheses. It is possible that bacterial density, not the *H. pylori* genotypes, stimulates mRNA synthesis of *TNF*-α. It is also possible that an increase in protein levels occurs without a significant increase in gene expression. The important finding is that, regardless of the pathway, *TNF*-α induces an intense inflammatory response that inhibits gastric acid production and suggests a worse prognosis.

In addition, we found that, contrary to results reported in the literature, *TNF*-α-308 G > A polymorphisms did not influence *TNF*-α mRNA expression in cases of gastric disease. In patients with postoperative sepsis, Baghel et al. [20] revealed that the AA homozygous genotype presents a greater capacity for producing *TNF*-α cytokines than other genotypes. Higuchi et al. [42] reported that the A variant of the *TNF-α*-308 polymorphism presented higher transcriptional activity and increased *TNF*-α cytokine production. Hosono et al. [43] found increased tumor necrosis factor receptor 1 expression in human colorectal adenomas, though *TNF*-α expression did not differ significantly.

These contradictory results may be explained, at least in part, by the fact that the Brazilian population is composed of a genetic miscegenation of various ethnic groups. This variation may influence allele and/or genotype frequencies and their correlations with gene expression. Furthermore, most studies evaluate protein levels of *TNF*-α, which are not necessarily associated with the mRNA levels of *TNF*-α. A recent study published by de Oliveira et al. [44] found results similar to ours. These authors reported that the polymorphic alleles of *TNF*-α (-308) did not have any effect on *TNF*-α or IL-10 expression levels. It is important to note that this study was performed on a Brazilian population and reinforces the hypothesis mentioned above.

## Conclusion

Ours results show that the presence of *Helicobacter pylori* induces a large increase in *TNF*-α expression in patients with gastritis, regardless of tissue inflammation, but after the tissue becomes neoplastic, the presence of bacteria did not influence expression. These results suggest that the *TNF*-α pathway may play an important role in the progression from gastritis to gastric cancer.

## Ethics committee approval

This study was approved by the Research Ethics Committee of Sacred Heart University (Universidade do Sagrado Coração) in Bauru, SP, Brazil (under registration n. 068/12).

## Consent

Written informed consent was obtained from all patients or their legal guardians for publication of this case report.
